# Cardiac Fibrosis in the Pressure Overloaded Left and Right Ventricle as a Therapeutic Target

**DOI:** 10.3389/fcvm.2022.886553

**Published:** 2022-05-06

**Authors:** Katharina Schimmel, Kenzo Ichimura, Sushma Reddy, Francois Haddad, Edda Spiekerkoetter

**Affiliations:** ^1^Division Pulmonary, Allergy and Critical Care Medicine, Department of Medicine, Stanford University School of Medicine, Stanford, CA, United States; ^2^Vera Moulton Wall Center for Pulmonary Vascular Disease, Stanford University, Stanford, CA, United States; ^3^Stanford Cardiovascular Institute, Stanford University, Stanford, CA, United States; ^4^Pediatric Cardiology, Stanford University, Stanford, CA, United States; ^5^Cardiovascular Medicine, Stanford University, Stanford, CA, United States

**Keywords:** fibrosis, extracellular matrix, cardiac fibroblast, cardiac function, left ventricle, right ventricle, pressure-overload, hypertension

## Abstract

Myocardial fibrosis is a remodeling process of the extracellular matrix (ECM) following cardiac stress. “Replacement fibrosis” is a term used to describe wound healing in the acute phase of an injury, such as myocardial infarction. In striking contrast, ECM remodeling following chronic pressure overload insidiously develops over time as “reactive fibrosis” leading to diffuse interstitial and perivascular collagen deposition that continuously perturbs the function of the left (L) or the right ventricle (RV). Examples for pressure-overload conditions resulting in reactive fibrosis in the LV are systemic hypertension or aortic stenosis, whereas pulmonary arterial hypertension (PAH) or congenital heart disease with right sided obstructive lesions such as pulmonary stenosis result in RV reactive fibrosis. In-depth phenotyping of cardiac fibrosis has made it increasingly clear that both forms, replacement and reactive fibrosis co-exist in various etiologies of heart failure. While the role of fibrosis in the pathogenesis of RV heart failure needs further assessment, reactive fibrosis in the LV is a pathological hallmark of adverse cardiac remodeling that is correlated with or potentially might even drive both development and progression of heart failure (HF). Further, LV reactive fibrosis predicts adverse outcome in various myocardial diseases and contributes to arrhythmias. The ability to effectively block pathological ECM remodeling of the LV is therefore an important medical need. At a cellular level, the cardiac fibroblast takes center stage in reactive fibrotic remodeling of the heart. Activation and proliferation of endogenous fibroblast populations are the major source of synthesis, secretion, and deposition of collagens in response to a variety of stimuli. Enzymes residing in the ECM are responsible for collagen maturation and cross-linking. Highly cross-linked type I collagen stiffens the ventricles and predominates over more elastic type III collagen in pressure-overloaded conditions. Research has attempted to identify pro-fibrotic drivers causing fibrotic remodeling. Single key factors such as Transforming Growth Factor β (TGFβ) have been described and subsequently targeted to test their usefulness in inhibiting fibrosis in cultured fibroblasts of the ventricles, and in animal models of cardiac fibrosis. More recently, modulation of phenotypic behaviors like inhibition of proliferating fibroblasts has emerged as a strategy to reduce pathogenic cardiac fibroblast numbers in the heart. Some studies targeting LV reactive fibrosis as outlined above have successfully led to improvements of cardiac structure and function in relevant animal models. For the RV, fibrosis research is needed to better understand the evolution and roles of fibrosis in RV failure. RV fibrosis is seen as an integral part of RV remodeling and presents at varying degrees in patients with PAH and animal models replicating the disease of RV afterload. The extent to which ECM remodeling impacts RV function and thus patient survival is less clear. In this review, we describe differences as well as common characteristics and key players in ECM remodeling of the LV vs. the RV in response to pressure overload. We review pre-clinical studies assessing the effect of anti-fibrotic drug candidates on LV and RV function and their premise for clinical testing. Finally, we discuss the mode of action, safety and efficacy of anti-fibrotic drugs currently tested for the treatment of left HF in clinical trials, which might guide development of new approaches to target right heart failure. We touch upon important considerations and knowledge gaps to be addressed for future clinical testing of anti-fibrotic cardiac therapies.

## Introduction

Reactive fibrosis is a process that accompanies cardiac remodeling in a wide range of heart diseases. Fibrotic remodeling of the heart affects the extracellular matrix (ECM), which is a three-dimensional elastic network of collagen fibers and non-structural proteins that embeds cardiomyocytes, vessels, cardiac fibroblasts, and immune cells, and represents the scaffold of the healthy heart. Depending on the etiology and pathological context, ECM remodeling can primarily affect the left or the right ventricle, respective atria, or all chambers of the heart at once.

**FIGURE 1:**
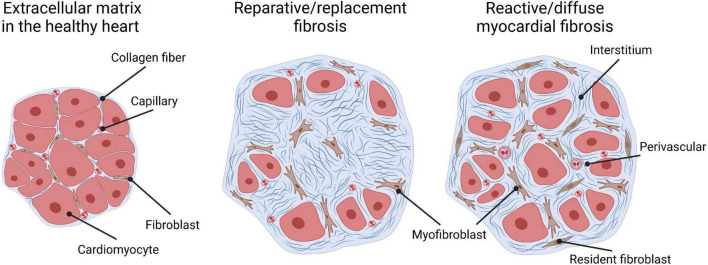
Types of cardiac fibrosis. The extracellular matrix in the healthy heart **(left)** is a three-dimensional network of collagen fibers that embeds cardiac cells such as cardiomyocytes, capillaries, and fibroblasts. “Replacement/reparative fibrosis” **(middle)** is visible as a collagen-based scar that is formed during a healing process and replaces dying cardiomyocytes after ischemic insults. “Reactive/diffuse myocardial fibrosis” **(right)** accompanies heart failure due to pressure overload and manifests as diffuse deposition of cross-linked collagens in interstitial and perivascular areas. Both patterns of fibrous deposits can be observed in patients with heart failure with reactive fibrosis, due to the formation of micro scars related to smaller foci of replacement fibrosis that are reflective of a loss of small numbers of cardiomyocytes.

Historically, cardiac fibrosis has been broadly categorized into two types: reparative, also called replacement fibrosis, and reactive fibrosis, also called diffuse myocardial fibrosis, given the fact that it manifests as excessive and diffuse deposition of ECM components in interstitial and perivascular areas (Figure 1). Replacement fibrosis is the result of a healing process after acute myocardial infarction (MI), or cardiomyocyte death of other causes. It is visible as a fibrotic scar triggered by ischemic cell death. Inhibition of this type of fibrosis in the acute phase of injury leads to local rupture of the ventricular wall and increased mortality (1–4). Reactive fibrosis on the other hand describes the diffuse deposition and cross-linking of collagens in interstitial and perivascular areas that occur in chronic cardiac conditions, as observed after MI in the remote zone of the remodeling myocardium, or in many non-ischemic heart conditions. An example resulting in reactive fibrosis would be HF associated with pressure overload due to long-standing hypertension or stenosis.

The notion that changes in the ECM of the human heart occur in cardiac disease is based on a very old idea and was first interrogated in 1957 (5). Even though this study documented no increase in collagen concentration in the LV in various conditions such as atherosclerosis, hypertrophy, and coronary occlusion, the hypothesis that the ECM might be affected in a way that disrupts cardiac function has been revisited time and again ever since and feeds a line of research that has been proliferative until today (3, 6–11). Over time, it became increasingly clear that not only ECM quantity (12–16), but also ECM quality should be taken into consideration. First, different collagen types occur in the heart that have different biophysical and physiochemical properties (17–19). Type I collagen represents approximately 85% of the total collagen proteins and builds thick, highly cross-linked fibers that are important for strength, whereas type III collagen confers elasticity to the interstitial network by forming flexible, thin fibers (6, 17, 19). In human hearts, both aging and pressure overload due to hypertension or aortic stenosis lead to an excess of the stiffer type I collagen over the more elastic type III collagen in the left ventricle (7, 9, 20, 21). Second, the degree of collagen cross-linking, which can be measured by taking the ratio of stiff insoluble to soluble collagen, rather than collagen amounts has been found to associate with LV stiffness in hypertensive patients with symptomatic (stage C) heart failure (22). Increased collagen cross-linking has also been found in endocardial biopsies from patients presenting with heart failure with preserved (HFpEF) or reduced (HFrEF) ejection fraction, and to correlate with increased myocardial stiffness (8, 23–25). Of note, increased collagen cross-linking by lysyl oxidase was found to be normalized after using the diuretic torsemide in a small number of patients with chronic HF (26) and resulted in normalization of LV chamber stiffness. Even though these effects might be collateral and a direct action of torsemide on cardiac fibrosis needs to be established, these data are suggestive of adverse ECM remodeling that might be taken into consideration as a potential candidate for direct therapeutic targeting.

In 1988, the cardiac fibroblast was identified to be the major source of collagen fibers, and the predominant type I collagen producing cell in the heart (27, 28). A line of extensive research uncovered the critical role of cardiac fibroblasts in initiation, modulation, and execution of ECM remodeling (29–31). It comes as no surprise that the cardiac fibroblast has been extensively studied as a potential target for ECM remodeling in the heart.

In this review, we focus on reactive fibrosis affecting the LV and the RV in chronic cardiac conditions and discuss the phenotypic and clinical consequences of adverse ECM remodeling in the pressure- overloaded heart. We reflect on therapeutic approaches aimed at improving detrimental effects of the fibrotic program of reactive fibrosis, and the potential relevance of preserving required functions of fibrosis. To facilitate anti-fibrotic drug development in the future, we identify open questions to be addressed by research, and potential considerations for clinical trial design.

## The Therapeutic Value of Targeting Fibrotic Remodeling in Failure of the Left or the Right Heart

**FIGURE 2:**
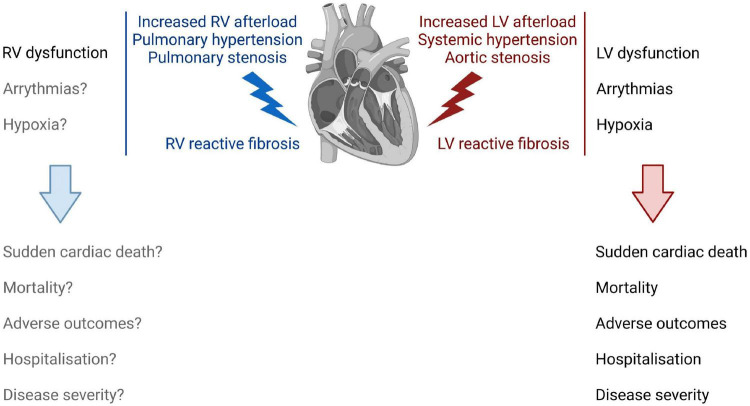
The impact of right and left ventricular reactive fibrosis on cardiac function, physiology, and clinical outcome. Increased LV afterload induces LV reactive fibrosis **(right)** that correlates with LV dysfunction, and increases the risk for arrythmias and for hypoxic conditions in the LV. Together, these changes in the LV increase the risk for sudden cardiac death, increase patient mortality, and associate with adverse outcomes, hospitalization, and disease severity. RV reactive fibrosis **(left)** following an increased RV afterload leads to increased RV stiffness and dysfunction. However, the role of fibrosis in the development of RV failure is not fully understood.

Despite extensive research, the exact role of cardiac fibrosis in heart diseases remains largely elusive. The extent to which fibrosis is a causal, contributing or an adaptive mechanism in the pathogenesis of cardiac conditions is likely context dependent, or co-exists, considering its heterogeneous nature as well as functional and phenotypic diversity (Figure 1). Reparative fibrosis, which locally replaces dying cardiomyocytes, might potentially be necessary to maintain the integrity of the heart (4, 29). Reactive cardiac fibrosis is hypothesized to be the result of an adaptive process that increases structural support to a higher workload for the heart. Accumulating evidence though suggests that reactive fibrosis correlates with changes in architecture, physiology, and function of the heart (7, 10, 11, 13, 30, 31), as well as disease severity and worse clinical outcome of patients with HF of various etiologies (32–35). Of note, it has been shown that fibrosis may precede the development of HFpEF (35), which might make a causative relation more plausible and warrants further investigations. In the following section, we therefore review existing evidence of the variable contribution of reactive fibrosis on left and right ventricular dysfunction, with a focus on pressure overloaded conditions (Figure 2). These observations might support the rationale for anti-fibrotic interventions to improve cardiac function and clinical outcomes in patients with reactive fibrosis.

### Extracellular Matrix Remodeling Impairs Left Ventricular Function and Physiology in Pressure Overloaded Conditions

HF is characterized by a progressive deterioration of LV function. Reactive fibrosis in the LV develops in chronic pressure overloaded conditions, such as hypertensive heart disease or aortic stenosis (Figure 2). Considering the importance of collagen cross-linking and accumulation of highly cross-linked collagen type I in myocardial stiffening, addressing the relationship between ECM remodeling and LV function and detailed phenotyping of these qualities was a major focus in fibrosis research during the past two decades. Collagen cross-linking determines the reversibility of experimental fibrosis (36), the stiffness of collagen fibers, and their resistance to degradation by matrix metalloproteinases (37). It can be measured in human myocardial biopsies or autopsies as well as experimental animal tissue by colorimetric and enzymatic assays, and after correcting the concentration of each form of collagen by the total amount of protein, collagen cross-linking is calculated as the ratio between insoluble and soluble collagen (38, 39). Mounting evidence suggests that the degree of collagen cross-linking, and an increase in the ratio of insoluble to soluble collagen, correlates with LV stiffness and diastolic dysfunction in hypertensive heart disease (8, 22, 23, 25) and aortic stenosis (40). Of note, therapeutic interventions for chronic HF such as the use of loop diuretic torsemide have been associated with an improvement in collagen cross-linking in the remodeled myocardium. In 2004, López et al. sought to determine if torsemide has anti-fibrotic effects on the failing heart beyond its effect on diuresis (41). Compared to furosemide, torsemide was able to improve LV diastolic function in chronic HF patients (42) and showed a lower mortality in the TORIC study (43), despite exerting similar renal effects (44, 45). These superior clinical effects of torsemide in patients with New York Heart Association functional class II to IV chronic HF were associated with lower collagen volume fractions (CVF) in myocardial biopsies and circulating carboxy-terminal peptide of procollagen type I (PIP), a biomarker for collagen type I synthesis (41). Based on these findings, the authors concluded that torsemide was able to reverse reactive fibrosis. In a pilot follow-up study, they demonstrated that torsemide modified collagen cross-linking by the enzyme lysyl oxidase (LOX) in patients with severe chronic HF that resulted in improvement of LV chamber stiffness that was more pronounced than in the furosemide treated group (26). Regardless of whether torsemide exerts a direct effect on the fibrotic responses or whether the observed effect is secondary to an improved heart failure management, these studies are evidence that reactive cardiac fibrosis can regress, along with improvement of LV function, which has important clinical implications for patients with HF. It should be noted that many of above studies have analyzed relatively small patient sample numbers. However, the collectivity of the resulting data might imply that interventions leading to regression of established reactive fibrosis could be successful in improving LV stiffness and dysfunction.

In addition to its impact on LV function, reactive fibrosis increases the risk of ventricular arrhythmias (46) (Figure 2). As such, all histological forms of cardiac fibrosis, diffuse interstitial fibrosis as characteristic for reactive fibrosis, patchy collagen deposition, or dense scars resulting from local healing processes provide substrate for arrhythmogenesis. Myocardial electrophysiologic consequences range from slower action potential propagation, re-entry initiation, promotion of after-depolarizations, to increased ectopic automaticity, and are greatly dependent on the architecture of fibrosis (47). Of note, reactive fibrosis is associated with ventricular arrhythmias independent of LV function in hypertensive heart disease (30). Anti-fibrotic therapies are therefore expected to have beneficial effects on ventricular arrhythmias in addition to beneficial effects on LV function.

Perivascular fibrosis, specifically, in addition to diffuse interstitial fibrosis, has implications for ischemia in the heart (Figure 2). For example, in patients with non-ischemic HF of various etiologies, coronary perivascular fibrosis associated with the impairment of coronary blood flow independently of interstitial fibrosis or cardiac function (48). Even though this was an observational study with a relatively small number of patients, these data might imply compromised coronary microcirculation and supply of nutrients and oxygen to the heart muscle in HF. Coronary microvascular rarefaction and fibrosis were also described in patients with HFpEF (49).

The hypothesis that reactive fibrosis might lead to hypoxic and dysfunctional collagen-encircled cardiomyocytes, was first tested 1995 in dogs with chronic HF induced by sequential intracoronary microembolizations (50). Even though confirming studies are required, results of previous work showed that reactive interstitial fibrosis in viable myocardium was inversely correlated to capillary density (capillary/fiber ratio), and positively correlated with an increased oxygen diffusion distance, suggesting that cardiomyocytes in LV regions with severe reactive interstitial fibrosis might be subjected to chronic hypoxia. Increased hypoxia in cardiomyocytes would eventually lead to more cell death, which would feed a vicious cycle in which dying cardiomyocytes were replaced by even more collagens in form of a reparative fibrotic process, leading to a further decline of LV function. In human studies, fibrosis and microvascular remodeling was assessed in 30 hearts with end-stage hypertrophic cardiomyopathy. Topological association of arteriolar abnormalities such as tunica media hypertrophy and intimal hyperplasia and severity of fibrosis was characterized in different areas (51). Further studies addressing the role of reactive fibrosis in generating a hypoxic environment for cardiomyocytes under pressure overloaded conditions are warranted.

As described above, reactive fibrosis is associated with distorted myocardial architecture, physiology, and cardiac function, and as such might play a critical role in the development of pressure-overload induced HF and adverse clinical outcomes in patients (Figure 2). Reactive LV fibrosis is implicated in the clinical course of HF in the following ways:

(*i*) *Reactive fibrosis plays a prognostic role in HF.* The severity of fibrosis assessed with cardiac magnetic resonance imaging (CMRI) correlates with a risk for sudden cardiac death, as well as cardiac and all-cause mortality in patients with non-high-risk hypertrophic cardiomyopathy (HCM) (33). Moreover, extracellular volume (ECV) associates with markers of disease severity in patients with HFpEF (32). It should be mentioned, however, that in terms of outcome, ECV did associate with outcome among CMR parameters, but only without adjustment for important clinical and invasive hemodynamic parameters in this study (32). However, a similar study found that fibrosis quantified by ECV CMR measures is prevalent in the LV of patients with HFpEF and associated with disease severity and adverse outcomes (35). In hypertensive heart disease, cardiac fibrosis associated with poor outcome and hospitalization with HF (52, 53). As such, reactive fibrosis was suggested to be a risk factor for sudden arrhythmic death in patients with heart disease of various etiologies (30, 46, 54, 55).

(*ii*) *Reactive fibrosis defines the response to treatment modalities in HF.* Weidemann et al. showed a correlation of severity of reactive fibrosis at the time of surgery and parameters of longitudinal systolic function with clinical outcome post-surgery by assessing standard and tissue Doppler echocardiography and late-enhancement cardiac magnetic resonance imaging (CMRI) at baseline and 9 months after aortic valve replacement in patients with severe aortic stenosis, as well as endomyocardial biopsies obtained during valve replacement surgery (56). These results are supported by Azevedo et al. who quantified fibrosis and LV function in patients with severe aortic stenosis by Gadolinium-enhanced MRI [contrast-enhanced (ce)-MRI] and picrosirius red staining of myocardial samples obtained at the time of aortic valve replacement, and a repeat ce-MRI 27 ± 22 months after surgery (34). To evaluate long-term survival, patients were followed for 52 ± 17 months. The amount of fibrosis inversely correlated with the extent of LV functional improvement, and directly associated with all-cause mortality post-surgery (34), suggesting that the degree of fibrosis predicts clinical outcome and long-term recovery after aortic valve replacement.

Although reactive fibrosis in the LV plays a prognostic role in HF and defines the response to treatment modalities, it represents one of many factors that change in adverse cardiac remodeling of the pressure overloaded heart. The extent to which reactive fibrosis contributes to the progression of HF compared to other possible key-players that can impact LV function, such as cardiomyocyte stiffness (57), or biomechanical LV wall stress (58, 59), remains to be addressed.

### Extracellular Matrix Remodeling Correlates With Right Ventricular Dysfunction in the Pressure Overloaded Right Ventricle

RV function is a key prognostic factor for patients with not only pulmonary arterial hypertension (PAH) (60) but also other subtypes of pulmonary hypertension (PH), such as group 2 PH (PH due to left heart disease) (61), group 3 PH (PH due to lung disease) (62), and also group 4 PH (chronic thromboembolic pulmonary hypertension) (63) (Figure 2). In fact, RV failure is the leading cause of death of patients with PAH (64). The development of fibrosis in the RV is a hallmark of RV failure and generally considered as a key player in the maladaptive process.

Several histology studies have reported mild but significant increase in RV fibrosis in patients with PAH (65, 66). CMRI studies reported that the volume of RV fibrosis strongly correlates with RV function, such as RVEF (67–69). Furthermore, increased biomarkers of collagen metabolism such as matrix metalloproteinase 9 (MMP-9) and tissue inhibitors of matrix metalloproteinase 1 (TIMP-1) are associated with disease severity in PAH (70). Nonetheless, the role of fibrosis in the development of RV failure and how that affects the RV function is not fully understood.

There is some evidence showing that RV fibrosis is related to end-diastolic wall stress (71). Using RV trabecular strips of pulmonary artery-banded (PAB) rodent models, Rain et al. showed that fibrosis-mediated stiffness was increased in animals with severe RV dysfunction and the ratio of collagen I/III expression was elevated (72). Kusakari et al. further reported that active tension and Ca^2+^ responsiveness was lower compared to sham animals in the RV papillary muscles (73).

However, it is still not clear whether targeting RV fibrosis is an ideal therapeutic target. A certain degree of RV fibrosis might be protective against mechanical stress by preventing excess RV dilatation and preserving the ventricular shape as it has been shown in the LV. Although RV stiffness is related to disease severity in PAH patients (65, 74), not only the amount of fibrosis but also the cardiomyocyte stiffness contributes significantly to RV stiffness particularly in the early stage of RV failure (72). Furthermore, RV functional recovery after mechanical unloading by lung transplantation has been reported, however, whether the functional recovery of the RV is related to reduced RV fibrosis is unknown. Studies in mice showed that cardiac fibrosis is in principle reversible once the RV afterload is normalized in a novel de-banding model where pulmonary arterial banding is reversed by using self-absorbable sutures (75).

As myocardial fibrosis both in the LV and the RV appears to be a useful indicator for long-term survival, it has implications not only as an important therapeutic target in the failing heart but also as a biomarker that could be assessed using a combination of biopsies, non-invasive imaging modalities, and circulating biomarkers.

## The Cardiac Fibroblast in Left and Right Ventricular Fibrosis

**FIGURE 3:**
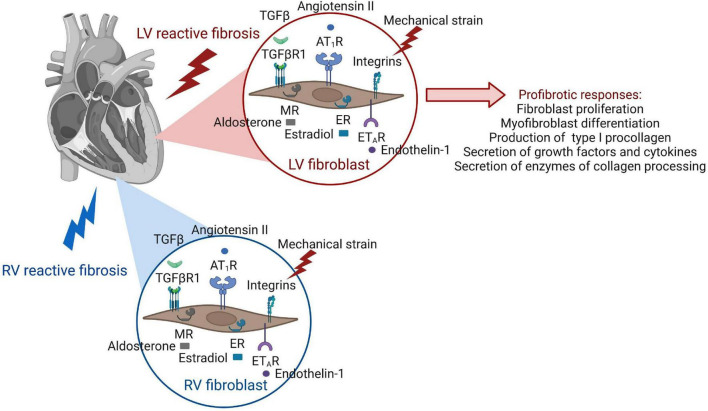
Receptor activation of cardiac fibroblasts leads to pro-fibrotic cellular behaviors. The same receptors of fibroblast activation are present in the fibroblasts of both ventricles, but involvement of signaling pathways and functional outcome of fibroblast responses have been mostly studied in the LV.

The cell biology of reactive fibrosis including mechanisms and pathways underlying fibroblast activation were most recently reviewed in great detail (76). We therefore provide a brief overview of newest insights on fundamental processes and key steps resulting in fibroblast behaviors that are responsible for an excessive deposition of collagens in the overloaded myocardium (Figure 3). Different stages of fibroblast activation have been described: Activation of resting cardiac fibroblasts to so called “activated fibroblasts” is accompanied by a high proliferation activity, periostin expression as well as secretion of collagen. Further differentiation of these activated fibroblasts into “myofibroblasts” changes the behavior and expression pattern of the cells which now express α-smooth muscle actin (α-SMA), less periostin, but more collagen than activated fibroblasts (77). A robust classification of fibroblast populations and activation stages requires the experimental ability to identify heterogeneous fibroblasts. With emerging technologies such as single-cell RNA sequencing (scRNAseq) we start to appreciate fibroblast heterogeneity present after pressure overload (78). However, it is currently unknown how and when different fibroblast populations or activation stages contribute to reactive fibrosis. Mechanisms of fibroblast activation described below are mostly derived from results generated before the advent of scRNA seq.

In the healthy adult mammalian heart, cardiac fibroblasts comprise around 20% of non-myocyte cells, without any significant changes in cell numbers or proliferative activities (79–81). Overloaded conditions lead to an accumulation of collagens, which might be due to the differentiation of activated fibroblasts to myofibroblasts, as well as an increase of cardiac fibroblast numbers in the heart. Historically, the latter event was thought to be dependent on endothelial-to-mesenchymal transition (EndoMT) and the recruitment of circulating hematopoietic progenitors to the diseased myocardium in various fibrotic heart conditions (82–84). This paradigm was revisited in 2014 by an in-depth analysis using refined fibroblast-lineage-tracing and demonstrated that accumulation of activated fibroblasts upon pressure overloaded conditions is neither attributed to EndoMT, nor the recruitment of hematopoietic progenitors, but to proliferation of activated resident fibroblast populations in the LV (81). Of note, the same study also found that αSMA, a marker of myofibroblasts, was only expressed by 15% of collagen1a1-labeled fibroblasts following pressure overload induction by TAC. These findings suggest that the accumulation of collagens seen in reactive fibrosis due to pressure overload might be the result of enhanced proliferation of activated resident fibroblasts, in addition to differentiation of activated fibroblasts to αSMA positive myofibroblasts.

The origin of fibroblasts in the RV during development differs from LV and RV. RV fibroblasts are derived exclusively from the epicardium *via* epicardium-to-mesenchymal transition, whereas fibroblasts in the LV are derived from both epicardium but also from endocardium *via* endothelial-to-mesenchymal transition (81). While fibroblasts in the LV under pressure overload have been studied extensively and shown to be derived by proliferation of resident fibroblasts (81), this has not yet been confirmed in the RV.

Myofibroblasts most recently have been shown to have protective effects in the pressure-overloaded LV (85). In the pressure overloaded LV after TAC, TGF-β/Smad3-activated cardiac myofibroblasts played an important protective role by preserving the ECM network, suppressing macrophage-driven inflammation, and attenuating cardiomyocyte injury. The protective actions of the myofibroblasts were mediated, at least in part, through Smad-dependent suppression of matrix-degrading proteases. It was suggested previously that a myofibroblast-mediated scar forms following cardiomyocyte death, resulting in microscopic scars when individual myocytes are lost (29). Indeed, myofibroblasts can engulf dead cells (86). This might imply that myofibroblasts may be the main effector cell responsible for reparative fibrosis not only following MI, but also in conditions of chronic overload. Further research is required to understand the exact contribution of different activation stages of resident cardiac fibroblasts to reactive fibrosis development and progression.

Collectively, this might result in new concepts supporting the rationale that targeting proliferation of activated resident cardiac fibroblasts in pressure-overloaded conditions rather than EndoMT, or the recruitment of hematopoietic progenitors might provide novel therapeutic opportunities, which warrant further investigations.

Factors driving pro-fibrotic behaviors that lead to the formation of diffuse myocardial fibrosis, such as increased fibroblast proliferation rates as well as collagen synthesis and secretion, are numerous in the diseased heart, and it can be anticipated that many of them occur at the same time. Cardiac fibroblasts have many receptors to respond to various stimuli (Figure 3).

Mechanical stress as occurring in pressure overload due to hypertensive heart disease and aortic stenosis can be sensed by cardiac fibroblasts by 2 classes of cell surface receptors, integrins, and discoidin domain receptors (87, 88). These receptors also mediate fibroblast interactions with the ECM and stimulate various cellular responses, such as fibroblast migration and proliferation, as well as differentiation into myofibroblasts.

Angiotensin II is not only expressed by the liver but can also be expressed by myofibroblasts. It can stimulate myofibroblasts in an autocrine manner, as well as most likely also other fibroblast populations in the heart, *via* binding to the G protein-coupled AT1 receptor (29, 89–91). This binding promotes fibroblast proliferative as well as procollagen I and III synthesis and secretion activities (29, 92, 93). Cardiac fibroblast proliferation is induced *via* mitogen-activated protein (MAP) kinases and the extracellular signal-regulated kinase (ERK) 1/2 that is activated by angiotensin II (93, 94). Procollagen I and III synthesis and secretion are mediated in this case by augmenting TGF-β/SMAD signaling pathways (29, 95). Of note, the profibrotic angiotensin II/AT1 receptor axis, has a counter-regulatory, fibro lytic axis in the myofibroblast and potentially also other fibroblasts in the heart, known as the ACE2-angiotensin (1–7)-Mas signaling axis (29). Angiotensin converting enzyme 2 (ACE2) is a homolog of ACE that is insensitive to ACE inhibitors and can hydrolyze angiotensin II into angiotensin (1–7) which binds to a Mas receptor to induce myofibroblast apoptosis through inhibition of antiapoptotic proteins (29).

TGF-β can signal through canonical, SMAD-dependent pathways, and non-canonical pathways. TGF-β-Smad2/3 signaling in activated resident cardiac fibroblasts has been identified as a key mediator of the fibrotic response in the pressure-overloaded LV (96). The canonical, SMAD-dependent pathway is mediated by the formation of a hetero-tetrameric complex between TGF-βRII and TGF-βRI molecules induced by TGF-β binding. The receptor complex then phosphorylates SMAD2 and SMAD3, which form a complex with SMAD4 in the cytoplasm (97, 98). The SMAD complex translocates to the nucleus and binds to SMAD binding elements to act as a transcription factor that leads to expression of collagen, CTGF, myofibroblast-associated proteins such as smooth muscle actin, periostin and IL11 (76, 99–102). In addition, TGFβ1 can trigger non-canonical, SMAD-independent signaling, that is mediated by TGFβ-activated kinase 1 (TAK1), mitogen-activated protein kinases (MAPKs) and Rho A (76). Importantly, in cultivated myofibroblasts isolated from hearts of patients with end-stage HF, inhibition of TGFβR1 kinase activity induced a dedifferentiation of myofibroblasts and a reduction of expression of profibrotic proteins, suggesting a certain plasticity of these cells (103).

Endothelin-1 (ET-1) is a vasoactive factor predominantly secreted by endothelial cells, but also by fibroblasts, cardiomyocytes, and macrophages. ET-1 can bind to ET receptors that are present on cardiac fibroblasts (104), thereby contributing to the development of myocardial fibrosis. Among many heart-independent functions, such as regulation of blood pressure through synergistic interactions with angiotensin II, proliferative effects of ET-1 in connective tissue disorders have increasingly been recognized (105).

Finally, it should be noted that both age and sex are factors that seem to mediate diffuse myocardial fibrosis (20, 106–111). With aging, increases in the production of fibrillar collagen were seen both in cardiac tissue of animals and human biopsies (110). In addition, collagen processing and maturation are altered, while collagen cross-linking due to advanced glycation end products (AGEs) increases, and collagen degradation becomes less effective. Molecular mechanisms associated with cardiac aging are inflammatory pathways (111), mitochondrial oxidative stress, insulin/insulin-like growth factor/PI3K pathway, adrenergic and renin angiotensin II signaling (RAAS), and nutrient signaling (112), all of which might directly act on the cardiac fibroblast to perpetuate reactive fibrosis. Female sex up to a certain age seems to have cardio-protective effects that might be explained by 17β-Estradiol-mediated differential estrogen receptor (ERα and ERβ) signaling in cardiac fibroblasts which regulate collagen I and III expression in the heart (113). In line, upon pressure-overload due to aortic stenosis, cardiac tissue of male patients was found to have higher expression of collagen types I and III than of female patients (106, 107).

In addition it should be noted that non-fibroblast cardiac cells such as cardiomyocytes, inflammatory, smooth muscle and endothelial cells present in both ventricles can also modulate cardiac fibroblasts by secreting factors directly impacting on fibroblast behaviors (76, 114–118).

## Extracellular Matrix Remodeling in the Left vs. Right Ventricle

**FIGURE 4:**
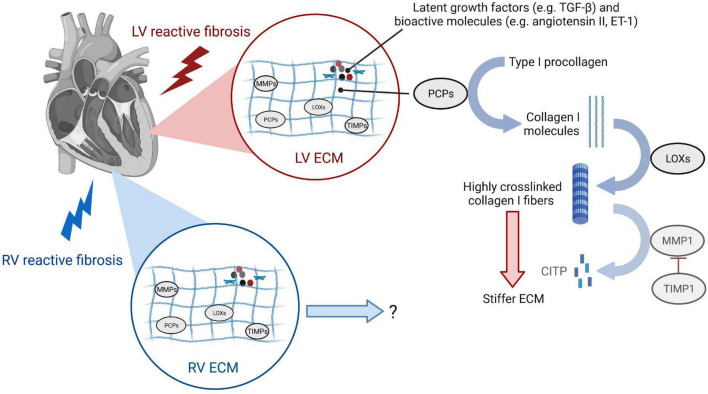
Changes of the extracellular matrix during reactive fibrosis leading to left ventricular stiffening. Enzymes responsible for collagen turnover are induced in both LV and RV reactive fibrosis. While studies show that collagen I cross-linking and deposition of highly cross-linked collagen I fibers impact LV function by stiffening the ECM, research is needed to understand ECM remodeling of the RV and functional consequences. Type I procollagen secreted by cardiac fibroblasts is processed to type I collagen molecules by procollagen carboxy-terminal proteinases (PCPs). Lysyl oxidases (LOXs) are enzymes that cross-link adjacent type I collagen molecules to highly cross-linked type I collagen fibers that stiffen the LV and have increased resistance to degradation by matrix metalloproteinase 1 (MMP1). MMPs, along with their inhibitors TIMPs, are responsible for ECM degradation to the carboxy-terminal telopeptide of type I collagen (CITP). While the same enzymes involved in collagen-turnover are induced in the pressure-overloaded RV, the dynamics of ECM deposition and maturation, and the extent of collagen cross-linking contributing to the severity of RV stiffness require further research.

The cardiac ECM provides a three-dimensional scaffold that is paramount for the healthy architecture, structure, mechanics, myocyte orientation, and transmission of contractile force of the heart (119–121). The ECM consists mostly of the structural type I and type III collagens that build the dynamic fibrillar network (122). In addition, it contains a wide range of glycoproteins, glycosaminoglycans, and proteoglycans, stored latent growth factors such as transforming growth factor-β (TGF-β) (123), bioactive molecules such as angiotensin II (124, 125) and endothelin (ET)-1 (126, 127), and enzymes catalyzing collagen maturation, processing, and degradation (128) (Figure 4). Pathologic remodeling of the ECM as occurring in reactive fibrosis is associated with changes in quantities, the composition of ECM components, and ECM quality such as collagen cross-linking. These quantitative and qualitative changes of the ECM can physically impair cardiomyocyte contractility and relaxation, and disturb electrical conductivity, as well as regional nutrient and oxygen diffusion. Outcomes of approaches that target some of the key modulators of these ECM changes, mostly tested for their effect on reactive fibrosis in the LV in experimental animals or patients, are discussed in section “Anti-fibrotic Therapies and Their Impact on Left or Right Ventricular Function in Animal Studies” and “Anti-fibrotic Drugs in Clinical Testing for Left Ventricle and Right Ventricle Heart Failure.” We here describe the role of important ECM modifiers in early development, progression, and degradation of diffuse myocardial fibrosis in the overloaded heart.

Excessive deposition of thick, highly cross-linked collagen type I fibers as seen for example in pressure-overloaded conditions (7, 9, 21) is the end-result of a highly complex process with many factors involved. Collagen synthesis by cardiac fibroblasts can be stimulated by mechanical strain, connective tissue growth factor (CTGF), TGF-β (31, 129), or angiotensin II (92). The cardiac fibroblast processes pro-collagen α-chains into triple helical pro-collagen, after a hydroxylation step of proline residues that takes place during the polypeptide elongation (128). The resulting 4-Hydroxyproline is a prerequisite for the triple helix formation and essential for its stability. Once collagen precursor type I procollagen is secreted by cardiac fibroblasts and myofibroblasts, enzymes called procollagen carboxy-terminal proteinases (PCPs), also produced by cardiac fibroblasts, convert these precursors into the mature collagen, type I collagen-forming microfibril molecules (130) (Figure 4). These molecules are then stabilized by inter-molecular covalent cross-links between polypeptide chains of adjacent fibrils that are catalyzed by the enzyme lysil oxidase (LOX). Resulting deposition of highly cross-linked collagen fibers leads to a stiffer ECM, which can activate cardiac fibroblasts and ensue a vicious cycle that promotes further progression of reactive fibrosis (87, 88). Elevated myocardial expression of PCPs, PCP enhancer 1 (PCPE1) which is a positive regulator of PCP, LOXs, and lysyl oxidase homolog 2 (LOXL2) has been observed in patients with HFpEF or HFrEF due to pressure overload, and associated with the severity of diffuse myocardial fibrosis determined by collagen deposition or collagen cross-linking (22, 23, 26, 131, 132).

Enzymes that degrade collagen fibers, such as matrix metalloproteinases (MMPs), and their inhibitors, tissue inhibitors of metalloproteinases (TIMPs) (Figure 4) have been proposed to play opposing roles in diffuse myocardial fibrosis (133). Clinical data are mixed and conflicting, which might be partly attributed to the fact that in some cases, mRNA or protein expression was measured without determination of actual enzymatic activities. A study analyzing the ratio between MMP-1 and its inhibitor TIMP-1 in cardiac tissue of patients with hypertensive HF by western blot and immunohistochemistry found that probands presenting with HFrEF had an excess of MMP1 relative to TIMP1, while the ratio in patients presenting with HFpEF was comparable with healthy individuals (10). On a histological level, patients with HFrEF had regionally diminished collagen deposits, but more perivascular and replacement fibrosis than their HFpEF counterparts. In addition, the MMP-1 to TIMP-1 ratio measured *via* enzyme-linked immunosorbent assay (ELISA) in serum was inversely correlated with the ejection fraction (EF) and directly correlated with LV end-diastolic diameter (LVEDD). Another interesting finding was that patients with aortic stenosis and HFrEF had higher levels of MMP1 and other MMPs and a lower level of TIMPs in the myocardium as compared to patients with HFpEF with the same etiology (134). The degree of collagen I deposition correlated with increased expression levels of MMPs, TIMP-1 and -2, and a decreased expression of TIMP-4 in this study, with the highest MMP expression levels together with gelatinolytic activities of MMP-2 as measured by zymography in the patient group with the lowest EF. The authors concluded that during the progression from compensated hypertrophy to HF, ECM turnover is characterized by the upregulation of MMPs and their inadequate inhibition by TIMPs (134). It was proposed that systolic dysfunction in patients with HFrEF due to hypertension or aortic stenosis might be caused by disruption of the mysial collagen scaffold that may facilitate cardiomyocyte slippage and loss of synchrony and synergy of cardiomyocyte contraction (135).

With in-depth reviews of the role of MMPs and TIMPs in cardiac extracellular matrix remodeling already existent in the literature (136), it should be noted that a consensus on whether reduced collagen degradation, or rather increased deposition might be the major contributor to overall collagen accumulation in the pressure-overloaded heart, has yet to be reached.

Although the role and composition of the ECM in the RV are considered to be similar to that of LV, few studies have investigated the difference between the LV and RV. A study using autopsy samples from patients who died of a non-cardiac cause showed that normal RV has higher collagen content than the LV: 7.4% in RV vs. 5.5% in LV (5). Another study using explanted donor hearts from patients with end-stage dilated cardiomyopathy and ischemic cardiomyopathy showed that the distribution of ECM proteins, such as laminin, fibronectin, and collagen I and IV, as well as MMPs differed between LV and RV (137). These differences are thought to be related to the fact that LV and RV have different embryological origin (138). Also, it could be related to the fact that the RV undergoes significant regression after switching from fetal circulation to postnatal circulation. Another striking fact is that the RV may be exposed to a fivefold increase in afterload, such as in the case of PAH, whereas the afterload increase the LV faces is usually less than twofold even in the most severe case of aortic stenosis. A study comparing the response of the RV and LV using PAB and transverse aortic constriction (TAC) mice models showed that several transcripts were differentially increased only in the pressure-overloaded RV (139). Studies like these are required to delineate potential differences between RV vs. LV reactive fibrosis due to pressure overload. However, limitations of this approach are that the relative pressure increase for a healthy RV after PAB is much higher than the relative pressure increase for a healthy LV after TAC, given that the healthy pulmonary circulation is a low-pressure system, which could lead to a differential gene expression. In general, most of our knowledge about the mechanism of RV fibrosis is extrapolated from LV research (140).

## Anti-Fibrotic Therapies and their Impact on Left or Right Ventricular Function in Animal Studies

As research uncovers the mechanisms involved in reactive fibrosis, new targets and strategies are identified. We here briefly highlight preclinical studies modulating some of the most relevant targets involving extracellular collagen turnover, and cardiac fibroblast responses in the LV and the RV.

Although numerous preclinical studies using animal models of PH (i.e., hypoxia-induced PH mouse model, monocrotaline rat PH model, and Sugen/hypoxia rat PH model) have shown a reduction of RV fibrosis in response to therapeutic interventions, most of these interventions also attenuated the pulmonary vasculopathy and decreased the pulmonary arterial pressure. Thus, it is not clear whether these interventions directly or indirectly reduced fibrosis by decreasing the RV afterload in these animal models. In this section, we will only discuss preclinical studies done in the pure RV pressure overload model (PAB) and the interventions aimed to directly target the RV.

### Angiotensin and Aldosterone

Systemic infusion of the pro-hypertensive hormone angiotensin II is a well-established and widely used *in vivo* preclinical model of LV reactive fibrosis due to pressure-overload mimicking systemic hypertension in patients (141). Given the gradual increase in pressure overload imposed on the heart of the animal as compared to models based on aortic banding, a relatively slowly progressing ECM remodeling can be achieved, which recapitulates human chronic disease (142). This led to a frequent use of this model of cardiac fibrosis, far beyond pre-clinical testing of Renin-Angiotensin-Aldosterone-System (RAAS) modulators. Interestingly, the profibrotic effect of angiotensin II could be seen upon chronic infusion of a low-dose angiotensin II, which did not increase systemic arterial blood pressures (143), and might therefore be mediated by direct effects of angiotensin II on cardiac fibroblasts (92, 93). Both Angiotensin 1 Receptor (AT1R) antagonists as well as Angiotensin Converting Enzyme (ACE) inhibitors have improved cardiac remodeling of the ECM and hypertrophy, together with reduction of arrhythmias, in pre-clinical testing (144–146).

Inhibition of the aldosterone pathway using mineralocorticoid receptor (MR) antagonists led to attenuated angiotensin II-induced fibrosis without affecting the blood pressure level in the mouse, halted progression of cardiac fibrosis and ameliorated myocardial global longitudinal strain in the hypertensive rat (147, 148).

As activation of the neurohormonal system has been also proposed as a key player in maladaptive RV failure, inhibition of the renin-angiotensin-aldosterone system by angiotensin receptor blockers (ARB) losartan (149) and Neprilysin inhibitor/ARB sacubitril/valsartan (150) has been tested and shown to reduce RV fibrosis without modifying RV afterload.

### Extracellular Collagen Processing

Considering the strong correlation between the extent of collagen cross-linking with impairment of cardiac function and with a poor prognosis in HF patients, the enzymes catalyzing covalent cross-links between polypeptide chains of adjacent fibrils, lysyl oxidase (LOX), and lysyl oxidase homolog 2 (LOXL2), could be of major interest as therapeutic target. However, it was suggested that on the one hand a healthy degree of collagen cross-linking is needed for the adequate formation of collagen fibers and on the other hand that a stable ECM might be beneficial in overloaded conditions. Reducing excessive LOX expression or restoring the balance in LOX activity might therefore be superior to the irreversible blockage of LOX enzymatic activity (151).

Direct inhibition of LOXL2 by specific neutralizing monoclonal antibodies in mice with cardiac hypertrophy 2 weeks after TAC-surgery led to the elimination of cardiac fibrosis progression and major improvements of LV function without impacting hypertrophy (132). Direct inhibition of LOX in a volume-overloaded rat model prevented LV dysfunction and improved profiles of collagen and MMPs/TIMPs (152).

Presumably indirect effects on LOX modulation were observed upon chronic blockade of the TGF-β type III receptor beta glycan using the peptide P144 in spontaneously hypertensive rats which led to a reduced expression of LOX and a decrease in collagen cross-linking (153).

In human TGF-β1-stimulated fibroblasts, EXP3179, a non-antihypertensive metabolite of angiotensin II antagonist losartan, reduced over-expression and enhanced activity of LOX (154). *In vivo*, chronic treatment with EXP3179 prevented LOX upregulation and collagen cross-linking without impacting the systemic blood pressure in a rat hypertensive model. Taken together, collagen cross-linking enzymes might be used as therapeutic targets of anti-fibrotic drugs.

### Transforming Growth Factor-β1

The role of the pro-fibrotic cytokine TGF-β1 was determined in animal studies using genetic and pharmacological interventions. TGF-β1 belongs to the TGF-β family of proteins, which have been described as the “master regulators” of fibrosis (155). Mice with inducible TGF-β receptor type R1/2 or SMAD3 deletions under the promotor of the myofibroblast marker periostin are protected against cardiac fibrosis and diastolic dysfunction in response to TAC (96). However, inducible overexpression of an inactive TGF-β type II receptor under the control of a metallothionein-derived promoter resulted in profound LV dilation and dysfunction in the pressure-overloaded mouse heart (156). Interestingly, cardiomyocyte-specific deletion of SMAD4, a central intracellular downstream mediator of TGF-β1 signaling, led to disturbances of systolic function in adult mice under basal conditions. Of note, canonical TGF-β signaling plays an important role in survival and contractility for cardiomyocytes during cell stress. In addition, TGF-β signaling can confer cardio protection not only in the pressure-overloaded (85), but also the infarcted myocardium (101). Therefore, caution is warranted when inhibiting this pathway therapeutically, in particular for long-term applications. On the other hand, heterozygous loss of TGF-β1 was shown to ameliorate myocardial fibrosis and LV compliance in aging mice, which explains their increased survival over the life span compared to wildtype control mice (157). Blocking of non-canonical TGF-β signaling by MAPK pathway inhibitors reduced ECM production in the renal fibroblast (158).

The TGF-β1 inhibitor pirfenidone, which is an approved drug for the treatment of idiopathic pulmonary fibrosis, showed anti-fibrotic effects in pre-clinical animal models of fibrosis across organs, namely lung, liver, LV, RV, and kidney (159). Pirfenidone not only inhibits synthesis and secretion of TGF-β1 by cardiac fibroblasts, but also their proliferation and migration activities, as well as differentiation into myofibroblasts (160). In various pre-clinical models of cardiac pressure-overload, pirfenidone improved LV function and fibrosis (161–163).

Reduction of RV fibrosis by pirfenidone has also been reported in the PAB animal model of RV pressure overload. Crnkovic et al. demonstrated a significant reduction of RV fibrosis by treating the animals with pirfenidone after RV fibrosis was established (i.e., 1 week after PAB); however, they did not observe an improvement in RV function (140). The authors speculated that this disconnection between reduction of RV fibrosis and RV dysfunction could be associated with the fact that RV dysfunction is not only driven by fibrosis but also strongly by the sarcomere impairment of the cardiomyocytes. On the other hand, Boehm et al. showed in the same animal model that activation of Bone Morphogenetic Protein signaling, a member of TGF-β superfamily, with FK506 was associated with reduction of fibrosis and improvement of RV function (164). They showed that FK506 reduced fibroblast proliferation and collagen production in a BMP-dependent fashion. Of note, the RV function of pirfenidone-treated animals were assessed after 3 weeks of treatment, while animals treated with FK506 were assessed after 7 weeks of treatment. This might suggest that the effect of fibrosis on RV function may differ during the development of pressure-overload induced RV failure. Further study is needed to elucidate the timing and duration of the therapeutic interventions in RV failure.

### Endothelin-1

ET-1 has been shown to be compartmentalized in the myocardial interstitium, and ET-1 uptake was enhanced in a swine model of chronic HF (127). Angiotensin II-induced cardiac hypertrophy, interstitial and perivascular fibrosis were found to be dependent on ET-1, independently from blood pressure (165), suggesting that ET-1 plays an important role in fibrosis development in hypertensive conditions.

The Endothelin receptor antagonist macitentan was shown to have anti-fibrotic effect in PAB rabbit model (165).

### Fibroblast Phenotypic Behavior

Newest approaches aimed at inhibiting pro-fibrotic responses of activated cardiac fibroblasts in a non-cellular-target-specific fashion. Discovery screens were based on the impact of drug candidates on proliferation rates (166, 167) and collagen type I expression of cardiac fibroblasts (167). Prominent anti-fibrotic effects of lead compounds identified correlated with improved LV function in two pressure-overloaded animal models with first promising safety data.

### Other Mechanisms Studied in the Right Ventricle

With the presumption that growth factor plays an important role in fibroblast proliferation, receptor tyrosine kinase inhibitors sunitinib and sorafenib have been examined in PAB rat model and have shown to reduce RV fibrosis (168).

Several other factors that been suggested to influence the RV adaptation. Activation of the nitric oxide (NO)/soluble guanylyl cyclase (sGC)/cyclic guanosine 3’5’-monophosphate (cGMP) pathway by the phosphodiesterase type 5 (PDE-5) inhibitor sildenafil (169) and the soluble guanylate cyclase activator riociguat (170) as well as activation of the prostacyclin pathway by iloprost (171) were first examined in PH animal models. When being tested in PAB models of isolated RV afterload, they were shown to reduce RV fibrosis suggesting an anti-fibrotic effect beyond their vasodilatory effect. The role of sex hormones, especially the protective role of estrogen and deleterious effect of androgen on RV function, has been proposed and castration (removal of testis) reduced RV fibrosis in PAB male mice (172). Another hallmark of maladaptive RV failure is a metabolic shift from glucose oxidation to glycolysis in the RV myocardium. Partial inhibition of fatty acid oxidation by trimetazidine and ranolazine reduced the collagen content in the RV of PAB rats (173). Another hallmark of RV failure, inflammation and oxidative stress was been targeted by a Nuclear factor kappa B (NF-kB) inhibitor (174), the antioxidant protandim (175), an apoptosis signal-regulating kinase 1 (ASK1) inhibitor (176), and p38 mitogen-activated protein kinase (MAPK) inhibitor (177) which resulted in a reduction of RV fibrosis, emphasizing the important but understudied interplay between inflammation and fibrosis—the immune system and cardiac fibroblasts.

## Anti-Fibrotic Drugs in Clinical Testing for Left and Right Ventricular Heart Failure

As discussed above, research efforts aimed at understanding the initiation and progression of adverse ECM remodeling have generated awareness of a panel of responsible key-players, some of which have subsequently been tested as therapeutic targets in humans. It should be noted that most anti-fibrotic therapies for the heart that seemed promising in experimental models have not been evaluated in patients, while the ones that underwent clinical testing mostly amounted to data that are limited or conflicting. We here discuss clinical testing of drugs that target extracellular collagen processing and/or cellular responses of the cardiac fibroblast (Figures 3, 4 and Table 1). No antifibrotic drug designed to specifically target ECM remodeling has reached clinical practice yet. However, some routinely used LV HF therapies have shown beneficial effects in this context, as outlined below in section “Renin–Angiotensin–Aldosterone System Inhibitors” and “Modulators of Extracellular Collagen Processing.”

**TABLE 1:** HF therapies and agents with the potential to modulate receptors present on cardiac fibroblasts or extracellular collagen processing in clinical testing for LV and RV overloaded conditions.

Cardiac condition	Target	Study	Agent	Patients (*n*)	Findings
LV overload	RAAS	(178)	ACE inhibitor lisinopril	35	Administration of lisinopril, but not hydrochlorothiazide for 6 months decreased CVF in endomyocardial biopsies of hypertensive patients
		(179)	ARB losartan	19	Administration of losartan for 12 months decreased CVF in endomyocardial biopsies of hypertensive patients with severe fibrosis, but not in patients with non-severe fibrosis
		(226)	ARB losartan	37	Administration of losartan but not amlodipine for 12 months decreased CVF in endomyocardial biopsies and serum biomarker PIP in patients with hypertensive heart disease
		RAAM-PEF	Aldosterone antagonist eplerenone	44	Administration of eplerenone for 6 months reduced biomarkers of collagen turnover in HFpEF patients with a history of hypertension
		Aldo-DHF	MR antagonist spironolactone	422	Administration of spironolactone for 12 months improved diastolic function but did not improve HF symptoms in HFpEF patients, mostly with a history of hypertension
		TOPCAT	MR antagonist spironolactone	3,445	Administration of spironolactone for 3.3 years decreased hospitalization for HF, but not death from cardiovascular causes, aborted cardiac arrest, or hospitalization for any reason in HFpEF patients, mostly with a history of hypertension
		(186)	MR antagonist spironolactone	40	Administration of spironolactone for 6 months decreased the rate of extracellular expansion but did not change myocardial extracellular volume measured by MRI in HFpEF patients
	Extracellular collagen processing	(26)	Diuretic torsemide	20	Administration of torsemide but not furosemide for 8 months decreased LOX protein expression and collagen cross-linking in endomyocardial biopsies of chronic HF patients
		(41)	Diuretic torsemide	36	Administration of torsemide but not furosemide for 8 months decreased CVF in endomyocardial biopsies and serum biomarker PIP of class II to IV chronic HF patients
		(131)	Diuretic torsemide	22	Administration of torsemide but not furosemide for 8 months decreased CVF and PCP activation in endomyocardial biopsies and serum biomarker PICP of chronic HF patients
		TORAFIC	Diuretic torsemide	155	Administration of torsemide or furosemide for 8 months had no effect on serum biomarker PICP in hypertensive patients with mild chronic HF
	TGFβ inhibitors	PIROUETTE	TGFβ inhibitor pirfenidone	80	Administration of pirfenidone for 52 weeks decreased ECV measured by MRI in HFpEF patients, mostly with hypertension
	ET-1 inhibitors	(205)	Dual ET A/B receptor antagonist enrasentan	72	Administration of enrasentan for 6 months increased LV-EDVI in asymptomatic patients with LV dysfunction
		EARTH	ET antagonist darusentan	485	Administration of darusentan for 6 months did not change LV-ESV at, measured by MRI in patients with LV systolic dysfunction
RV overload	RAAS	STAR-HF	MR antagonist spironolactone	30 estimated	Ongoing; administration of spironolactone for 12 weeks will be assessed by biomarkers of fibrosis and T1 weighted MRI in patients with right HF

*ACE, angiotensin converting enzyme; CVF, collagen volume fraction; ARB, angiotensin receptor blocker; HFpEF, Heart Failure with preserved ejection fraction; MR, mineralocorticoid receptor; MRI, magnetic resonance imaging; LOX, lysil oxidase; PIP/PICP, procollagen type I carboxy-terminal peptide (biomarker of collagen type I fiber synthesis); TGFβ, Transforming Growth Factor-β1; ET, endothelin-1; LV-EDVI, left ventricular end diastolic volume index; LV-ESV left ventricular end-systolic volume.*

### Clinical Trials Targeting Fibrosis in Left Ventricular Heart Failure

#### Renin–Angiotensin–Aldosterone System Inhibitors

Clinical evidence indicates that the mainstay therapy for LV systolic HF, inhibiting the RAAS axis, targets diffuse myocardial fibrosis. As outlined in section “Extracellular Matrix Remodeling in the Left vs. Right Ventricle,” cardiac fibroblasts express an AT1 receptor and respond to angiotensin II binding by ECM production and cellular proliferation (90, 92, 93, 124). In line, the ACE inhibitor lisinopril or the ARB losartan were associated with reducing the extent of reactive fibrosis together with LV stiffness, and improving LV diastolic dysfunction, when given to small cohorts of patients with hypertensive heart disease (178, 179).

Mineralocorticoid receptor (MR) antagonists are another class of established drugs for the treatment of LV systolic HF. Aldosterone can augment angiotensin II signaling, and also induce cardiac fibroblast proliferation independently of angiotensin II signaling, when binding to the MR *via* upregulation of MAPK pathways (180, 181). The double−blind clinical trial TOPCAT (Aldosterone Antagonist Therapy for Adults With Heart Failure and Preserved Systolic Function) testing the MR antagonist spironolactone in 3,445 HFpEF patients, did not significantly reduce the incidence of death from cardiovascular causes, cardiac arrest, or hospitalization (182). However, a subgroup of selected patients, many with atrial fibrillation and elevated heart failure biomarkers, showed a clinical benefit of spironolactone administration (183). While the rationale for this trial was based on the potential of MR antagonists to reduce fibrosis, cardiac fibrosis was not a studied end-point. In addition, aldosterone or MR antagonists led to improved tissue relaxation and positive changes in markers of collagen turnover in patients with HFpEF (184, 185). Recent studies applying CMR−derived measures of diffuse myocardial fibrosis as a primary end point demonstrated spironolactone-mediated decrease in the rate of extracellular expansion, with a decrease in the masses of cellular and extracellular myocardial compartments, in patients with HFpEF (186). Above therapies are safely used in the clinic and have the ability to target the AT1 or the MR axis in cardiac fibroblasts. However, some fibrotic burden still remains upon treatment, and given the prognostic role of fibrosis, this might contribute to the lack of a mortality benefit or reduction in hospitalization within HFpEF patients.

#### Modulators of Extracellular Collagen Processing

Diuretics are in clinical use to unload the heart. In small studies with patients with hypertensive HF, addition of the loop diuretic torsemide to conventional HF therapy led to beneficial modulation of extracellular collagen processing that associated with improvements in ECM quality and LV function (26, 41, 131). A decreased activity of the mature collagen type I-forming enzyme PCP and therefore less collagen deposition, as well as decreased expression of the collagen cross-linking catalyzing enzyme LOX and therefore less collagen cross-linking was observed after torsemide treatment. This was true only for the loop diuretic torsemide, but not furosemide. However, in a multi-center study of 155 hypertensive patients with chronic HF, the TORAFIC study, there were no significant differences between torsemide or furosemide groups in terms of changes in the serum procollagen type I carboxyterminal peptide (PICP), a biochemical marker of collagen type I fibers synthesis and deposition (187). It should be noted that the TORAFIC study did not assess fibrosis with histological or imaging modalities. In addition, the patient population had less severe HF compared to the preceding studies in smaller cohorts of hypertensive HF patients. Further studies are required to assess if it would be beneficial to select patients who might benefit from treatment with torsemide over furosemide. Nevertheless, long-term administration (around 10 years) of loop diuretics seemed to be safe.

Collectively, even though exact mechanistic studies are missing, modulation of ECM quality *per se* might represent a promising avenue for fibrosis treatment in the future and should be tested in large randomized controlled clinical trials. Of note, inhibition of LOXL-2 using the humanized monoclonal antibody simtuzumab has already been tested in phase II clinical trials in patients with hepatic or pulmonary fibrosis, demonstrating the feasibility direct targeting of collagen cross-linking (188, 189).

#### Transforming Growth Factor-β1 Inhibitors

Myocardial and serum levels of the profibrotic cytokine TGF-β1 are elevated in patients with HF due to hypertension or aortic stenosis (190, 191). The small molecule pirfenidone is orally bioavailable, and one of the most extensively studied inhibitors of the profibrotic cytokine TGF-β1. Pirfenidone reduces the synthesis and release of TGF-β1 in cardiac fibroblasts *in vitro*, as well as expression of TNF-α and PDGF, and is licensed for the treatment of idiopathic pulmonary fibrosis (160, 192, 193). In patients with pulmonary fibrosis, pirfenidone was shown to be safe and well tolerated, with infrequent and mild gastrointestinal and skin-related adverse events (194–197). Pirfenidone recently underwent a randomized, double-blind, placebo-controlled phase II PIROUETTE (Pirfenidone in patients with heart failure and preserved left ventricular ejection fraction) trial for HF, with CMR-assessed diffuse myocardial fibrosis as one of the study endpoints (198). Of note, patients with HFpEF were stratified based on fibrosis burden at baseline using ECV on CMR, based on the histologically-backed observation that regression of myocardial fibrosis was most pronounced in patients with the greatest burden of fibrosis at baseline (178, 179, 199). Given the fact that pirfenidone does not have any hemodynamic effects, results from the PIROUETTE trial might be of great interest also from a mechanistic perspective. First modest beneficial results were presented by Dr. Christopher Miller at the American College of Cardiology Virtual Annual Scientific Session (200), May 17, 2021. Pirfenidone led to a modest reduction in myocardial fibrosis without impacting diastolic function or 6-min walk distance (201). Potential deleterious effects, especially for long-term treatment regimens using pirfenidone, could arise due to the cardio protective side of TGF-β signaling described in section “Transforming Growth Factor-β1,” and should be closely monitored for in future studies.

#### Endothelin-1 Inhibitors

While the dual endothelin receptor subtype A and B antagonist bosentan prevented fibrosis of various organs in animal models (202), the majority of clinical studies show that inhibition of the vasoconstrictor peptide endothelin-1 might not be the ideal anti-fibrotic strategy for the heart. For the treatment of pulmonary hypertension, the dual endothelin (ET) A and B inhibitors bosentan and macitentan, as well as the mono ET A inhibitor ambrisentan, are FDA approved. Initially, a small study in 36 men with severe HF showed promising results on systemic and pulmonary hemodynamics upon additional administration of bosentan (203). However, subsequent clinical testing of various endothelin receptor antagonists were neutral or showed harmful effects (204, 205). For example, administration of enrasentan resulted in adverse cardiac remodeling assessed by CMR parameters of LV end-diastolic volumes in 72 patients with asymptomatic LV systolic dysfunction (205). In a double-blind, placebo-controlled trial testing five different doses of darusentan, no improvement in cardiac remodeling, clinical symptoms or outcomes based on CMRI endpoints was shown in 642 patients with chronic HF (204). The general conclusion drawn was that endothelin-1 blockade might not be useful as an add-on treatment.

### Clinical Trials Targeting Fibrosis in Right Ventricular Failure

Currently, there is only one clinical trial (STAR-HF: NCT03344159) assessing RV fibrosis as one of the outcomes. This trial is a phase 4 clinical trial investigating the effect of the mineralocorticoid receptor antagonist spironolactone in patients with chronic RV failure caused by either PH or cardiomyopathy. RV fibrosis will be assessed *via* T1 weighted MRI and serum biomarkers. There are no clinical trials that are directly targeting RV fibrosis or are examining the feasibility of anti-fibrotic therapy in RV failure.

## Future Directions

Despite pre-clinical successes, clinical testing of anti-fibrotic compounds has been unsuccessful. Endeavors to develop therapeutic approaches for reactive myocardial fibrosis go hand in hand with fundamental questions, such as (*i*) *Should therapeutic strategies block chronic maladaptive forms of fibrosis, while preserving its adaptive function to confer chamber stability, or locally replace dying cardiomyocytes upon overloaded conditions?* (*ii*) *Is established reactive myocardial fibrosis reversible?* To aid in the development of effective and safe therapeutic strategies, we are discussing these important considerations in the following section.

### The Potential Necessity to Preserve Adaptive While Targeting Maladaptive Functions of Fibrosis in the Overloaded Heart

Myocardial fibrosis in pressure overloaded conditions is very complex and given its heterogeneity in functional profiles, there is a call for a better definition, phenotyping, and quantification of the different types of fibrosis that exist in HF (206) (Figure 1). Toward this end, studies using invasive biopsy and non-invasive imaging show that in severe aortic stenosis, three main subtypes of fibrosis co-exist in the LV: pronounced fibrotic endocardial thickening, micro scars in the sub-endo myocardium resulting from reparative fibrosis, and diffuse interstitial fibrosis corresponding to reactive fibrosis (207). In other words, reparative fibrosis seems to take place locally to replace dying cardiomyocytes, whereas at the same time reactive fibrosis leads to adverse remodeling resulting in worse LV function and clinical outcome for the patient. This raises the question whether long-term antifibrotic intervention that also targets the potentially required reparative fibrosis in form of micro scars might be beneficial. Pre-clinical studies indicate that early stages of ECM remodeling of the pressure-overloaded, hypertrophied non-human primate heart are accompanied by reactive fibrosis without cardiomyocyte death, while in later stages a certain degree of reparative fibrosis is present (208). If this timeline was translatable to human ECM remodeling, clinical trials testing new anti-fibrotic therapies would need to be designed with optimal timing in mind. In a small systematic histological and histometric study of 30 hearts with end-stage hypertrophic cardiomyopathy, harvested at time of transplant, replacement fibrosis was the most prevalent form of fibrosis which in total occupied almost over a third of the LV (51). Antifibrotic therapy therefore might not necessarily be beneficial in end-stage heart failure when replacement fibrosis instead of reactive fibrosis is most prevalent. Taking into consideration that reactive fibrosis might lead to hypoxic conditions that favor cardiomyocyte death, anti-fibrotic interventions at earlier timepoints might be indicated. This would help prevent local replacement fibrosis and micro scar development, a known substrate for arrhythmias. As reactive fibrosis initially might be an important adaptive mechanism to add to chamber stability and prevent dilation of the ventricle, it is important to characterize at which time-point and which amount of reactive fibrosis becomes maladaptive. In general, any additional management of underlying etiologies to reduce the cardiac afterload would be paramount.

### How Do We Assess and Quantify Fibrosis in Humans Longitudinally With Therapeutic Intervention

A combination of imaging techniques and circulating biomarkers indicating activity of reactive myocardial fibrosis in real-time would be useful to guide timing of anti-fibrotic interventions, and to monitor efficacy. Latest imaging techniques employ CMRI as a non-invasive assessment tool not only of cardiac structure and function, but also of tissue characteristics. CMRI allows direct detection of fibrotic patches as seen in large post-infarct scars *via* late-gadolinium enhancement (LGE), and indirect assessment of diffuse interstitial fibrosis by T1 mapping of the myocardial wall, including native T1 differentiating normal from infarcted myocardium, post-contrast T1, and extracellular volume fraction (ECV) (209). However, clinical studies validating ECV measurements with quantification of histological collagen deposition in biopsies seem conflicting (207, 210, 211). In addition, LGE techniques are qualitative and not able to visualize replacement fibrosis occurring in the overloaded heart, which can occupy very small regions and is not associated with a large scar as present following myocardial infarction. Further limitations of CMRI-based assessments are a lack of sensitivity in detecting early stage disease, as well as of the ability to capture active fibrogenesis of reactive fibrosis. As such, molecular imaging of cardiac fibrosis could have the potential to overcome all these obstacles (212), both for the LV as well as the RV. Molecular imaging can sensitively quantify specific fibrotic pathways by using small molecules, peptides, or antibodies that are tagged with an imaging reporter, and that bind to a protein, receptor, or fibrotic process of interest (213). The molecular imaging probes can then be visualized with positron emission tomography (PET), single-photon emission computed tomography (SPECT), or MRI. Molecular imaging has already successfully reached the clinics for applications related to various fibrotic lung diseases, thanks to pioneering studies conducted within the last two decades (214–216). For the fibrotic heart, development of this technology is lagging behind and until now it has been used to demonstrate focal scarring after myocardial infarction in pre-clinical (217–220) and preliminary clinical studies (221) by tracing myofibroblasts, or collagen type I. Recent advances demonstrated feasibility of detecting diffuse interstitial myocardial fibrosis using specific collagen probes in the β2-AR (β-2-adrenergic receptor) overexpressing mouse model *in vivo*, and in ischemic human heart specimens (222). Importantly, specific tracers directed toward molecular targets that allow for a differentiation of pro-fibrotic responses associated with the different stages of a wound healing processes, and other fibrotic processes that also occur in a pressure-overloaded condition, such as ECM accumulation and cross-linking, do exist (212, 213). Further research to validate their usefulness in the overloaded heart is warranted.

### Evidence of Reversibility of Reactive Cardiac Fibrosis

Pre-clinical and clinical histological evidence suggests that in general, established reactive myocardial fibrosis is able to regress in the pressure-overloaded LV, even at later stages (178, 179, 223–225). Regression of fibrosis was evident in these cases after unloading the heart by treating underlying hypertension. Experimental models of hypertensive cardiac fibrosis include the angiotensin II infused mouse, with normalization of blood pressure achieved by cessation of angiotensin II infusion that led to an incomplete resolution of myocardial fibrosis with concomitant improvement of cardiac function (223). In Sprague-Dawley and in spontaneously hypertensive rats, regression of collagen deposition occurred after treatment with angiotensin-converting enzyme (ACE) inhibitors, and in cases that included cardiac functional measurements, fibrosis regression was seen together with an improvement of abnormal myocardial diastolic stiffness (224, 225). In a study using a small number of patients with hypertensive heart disease, ACE inhibition with lisinopril regressed myocardial fibrosis on endomyocardial biopsy that was accompanied by improved LV diastolic function (178). Interestingly, the ARB losartan induced regression of myocardial fibrosis that was associated with a reduction of myocardial stiffness in hypertensive patients with severe fibrosis, but not with non-severe fibrosis, as assessed by collagen volume fraction (CVF) quantification in endomyocardial biopsies in a small study (179). This finding might imply that patients with hypertension might benefit from treatment stratification that includes assessment of their fibrotic status. Of note, normalization of blood pressure did not prove to be effective in reversing established cardiac fibrosis in all cases. For example, normalization of blood pressure using the calcium channel blocker amlodipine failed to reduce CVF in endomyocardial biopsies, and serum levels of PIP in hypertensive patients (226). *In vitro* studies using rat cardiac fibroblasts demonstrated that cardiac fibroblasts expressed an angiotensin II receptor that controls proliferative activities and is sensitive to the antagonist losartan (91, 93), suggesting that fibrosis regression by ACE inhibitors and ARBs might at least in part be mediated by direct actions on cardiac fibroblasts.

Clinical data on the reversibility of reactive fibrosis after unloading the LV from pressure by replacing the aortic valve in the setting of aortic stenosis seem reaffirming. In a study utilizing CMRI and CVF assessment in biopsies in patients with severe aortic stenosis, aortic valve replacement led to regression of reactive fibrosis, but not replacement fibrosis, that was accompanied by structural improvements and amelioration of diastolic dysfunction after 1-year post-surgery follow-up (227). In line, a study that included asymptomatic mild, moderate, and severe aortic stenosis patients, as well as symptomatic patients who underwent aortic valve replacement, showed that reactive myocardial fibrosis regressed, while midwall LGE was irreversible post valve replacement (228). It should be noted that a third study concluded that myocardial fibrosis is not reversible 9 months after aortic valve replacement (56). However, histology was only assessed at time of surgery, and CMRI included LGE for assessment of reparative fibrosis but not T1 mapping and ECV for reactive fibrosis. The results of this study might be congruent with subsequent studies as noted above, concluding that replacement fibrosis might not be reversible. In contrast, reactive fibrosis can regress in the LV upon unloading of pressure-overload due to aortic stenosis.

In other settings, cardiac fibrosis might be not reversible. For example, mechanical volume and pressure unloading of the heart by left ventricular assist devices (LVAD) as a means to bridge time until a heart transplant becomes available, or as a final treatment option for end-stage HF, sometimes is followed by cardiac recovery so that the LVAD can be weaned off in some cases. Interestingly, pre-clinical studies demonstrated that replicating mechanical unloading in a rat model of TAC by heterotopic heart transplantation led to complete regression of cardiomyocyte hypertrophy, while fibrotic remodeling was significantly exacerbated (229). The authors concluded that this result might explain the low rates of LVAD removal in the clinics. As a matter of fact, reports describing LVAD explantation after successful myocardial recovery under LVAD therapy are rare (230, 231). Moreover, clinical data using a small number of patients showed persistence of cardiac fibrosis after 8 months of unloading using a LVAD (232). Conflicting results suggested that reduced MMP activity diminished damage to the ECM and contribute to LV functional recovery after LVAD support (39). Further studies with larger patient numbers might be required to reach conclusive results.

Mechanistically, reversal of reactive cardiac fibrosis likely includes degradation or modification of the fibrotic extracellular matrix, and changes in cellular behaviors of activated fibroblasts and myofibroblasts such as proliferation and collagen I synthesis, apoptosis, senescence, dedifferentiation, and reprogramming. Further research would be needed to understand mechanisms of fibrosis resolution, which might benefit from newest technical developments, such as single cell RNA sequencing (scRNAseq), or molecular imaging techniques.

Even though regression of reactive myocardial fibrosis is possible in some scenarios as mentioned above, complete fibrosis resolution and regaining of healthy state levels have never been achieved in humans. This observation supports the notion that further regression would require the development of additional fibrosis-resolving treatments. However, as reactive fibrosis develops in response to a mechanical stimulus such as pressure-overload it may play additional important adaptive roles by providing structural stability. Unless there is evidence of ongoing, unrestrained, and non-beneficial fibrosis at some point in the cardiac remodeling process, there might be therefore also potential negative consequences of inhibiting fibrosis. In any case, treating the underlying condition in parallel by for example reducing RV pressure-overload or aortic stenosis would be paramount.

## Conclusion

Reactive fibrosis in the pressure overloaded LV and RV accompanies cardiac remodeling that leads to heart failure. Most studies aimed at understanding the impact of reactive fibrosis on cardiac function, physiology and patient outcomes have been conducted in the LV. A limitation of probably all human correlational studies is the small patient number and occasional inconsistencies in readouts that range from histology, imaging modalities to fibrosis-biomarkers. In addition, up to one-third of HFpEF patients have normal measures of myocardial fibrosis, which might call for stratification for individuals that might benefit from anti-fibrotic therapies. Lastly, timing of anti-fibrotic drug administration might be an important factor to be considered for clinical trial designs to optimize efficacy and safety. Non-invasive imaging could be used to guide optimal timing and monitor treatment response. CMRI can be used to determine reactive fibrosis *via* T1 mapping and ECV assessment and captures existing ECM deposits in the heart. Molecular imaging would additionally enable visualization of active fibrogenesis of reactive fibrosis occurring in real-time. In addition, T1 mapping would allow reactive fibrosis to be graded, and the question if there is a point of no return for reversal of reactive fibrosis could be answered.

Fibrosis research has been hampered by technical limitations affecting both *in vitro* and *in vivo* experiments. The mayor limitation of *in vitro* studies is the lack of healthy fibroblasts in a culture dish due to immediate differentiation into myofibroblasts that is induced by the strain of the plastic surface. This imposes difficulties not only in understanding differences in the healthy vs. activated fibroblast, but also dissecting the role of myofibroblasts vs. proliferating resident fibroblasts in myocardial remodeling. Cardiac fibroblast markers used *in vivo* mostly lack specificity and for that reason, cardiac fibroblast-specific ablation of factors of interest remains challenging. However, it should be noted that emerging newer technologies, such as scRNA seq, might help in these quests. Most recently, a vast heterogeneity in cardiac fibroblast populations has been uncovered using scRNAseq (86), and differences in their functions need to be addressed in future studies. Time-dependent changes in fibroblast populations or states in the pressure-overloaded LV or RV and their functional contributions to reactive fibrosis development, progression or regression should be determined. In addition, other cell types involved in these processes might be uncovered. This will be helpful in guiding drug development, and timing of administration.

Most of our perception on reactive fibrosis stems from studies focused on the LV. Further research is needed to understand the contribution of reactive fibrosis in the RV with regards to RV dysfunction, arrhythmias, and creation of hypoxic conditions. In addition, RV fibrosis as predictor of survival of patients is currently unknown. RV biopsies of the RV free wall would put patients at risk for perforation, therefore the use of non-invasive imaging and biomarkers to assess reactive fibrosis in addition to the meticulous study of human heart tissue at the time of transplant or surgery, would be the first-line modalities to address these questions.

Despite all the above-mentioned challenges in fibrosis research, the observation that fibrosis regression is possible, is promising and a fascinating area to study. Understudied research areas include the process of intracellular collagen degradation, namely phagocytosis of macrophages and cardiac fibroblasts (233) as well as the modulation of collagen cross-linking, as a means to reverse fibrosis. Given the structural stability conferred to the heart by reactive fibrosis, therapeutic strategies that induce fibrosis resolution should be accompanied by management of the underlying etiology, such as interventions that relieve the afterload. Collaborative efforts across disciplines including cell biology, mouse genetics, imaging, cardiology, and pharmacology will be required to help unveil mechanisms and dynamics of reactive fibrosis and translate them into therapeutic approaches that can be tested and ultimately used in clinical practice.

## Author Contributions

KS and ES conceptualized the review. KS and KI wrote the manuscript. KS created the figures and table. ES, SR, and FH critically reviewed and provided feedback and guidance. All authors contributed to the article and approved the submitted version.

## Conflict of Interest

The authors declare that the research was conducted in the absence of any commercial or financial relationships that could be construed as a potential conflict of interest.

## Publisher’s Note

All claims expressed in this article are solely those of the authors and do not necessarily represent those of their affiliated organizations, or those of the publisher, the editors and the reviewers. Any product that may be evaluated in this article, or claim that may be made by its manufacturer, is not guaranteed or endorsed by the publisher.
